# Thymoquinone Ameliorates Gut Epithelial Injury by Suppressing the JNK Signaling Pathway Based on Its Anti‐Oxidant Property

**DOI:** 10.1002/fsn3.70113

**Published:** 2025-03-24

**Authors:** Kaitong Jia, Lei Wu, Ziru Li, Tian Wei, Tingting Fan, Guiran Xiao

**Affiliations:** ^1^ China Light Industry Key Laboratory of Meat Microbial Control and Utilization Hefei University of Technology Hefei China; ^2^ School of Food and Biological Engineering Hefei University of Technology Hefei China; ^3^ Anhui Provincial International Science and Technology Cooperation Base for Major Metabolic Diseases and Nutritional Interventions Hefei University of Technology Hefei China; ^4^ Engineering Research Center of Bio‐Process Ministry of Education, Hefei University of Technology Hefei China

**Keywords:** *Drosophila*, JNK, ROS, thymoquinone, ulcerative colitis

## Abstract

Ulcerative colitis is one of the most common sorts of inflammatory bowel disease. This study investigates the protective effects of thymoquinone against sodium dodecyl sulfate (SDS)‐induced intestinal damage and elucidates the underlying mechanisms using the *Drosophila melanogaster* model of ulcerative colitis. We found that *Drosophila* fed thymoquinone from larval to adult stages were resistant to SDS injury in adulthood. Thymoquinone pretreatment significantly restored the abnormal behaviors and intestinal morphological defects in *Drosophila* exposed to SDS. Moreover, thymoquinone protected the intestinal barrier function by inhibiting the overactivated c‐Jun N‐terminal kinase (JNK) pathway in the intestine induced by SDS. Further studies indicated that thymoquinone inhibits the JNK pathway by reducing intestinal reactive oxygen species (ROS) levels. This research provides novel pathological and mechanistic insights into the potential application of thymoquinone in developing functional foods or natural medicines, highlighting its significance in treating ulcerative colitis.

## Graphical abstract

A model explains thymoquinone's effect on ulcerative colitis. SDS in the intestines induces ROS, activating JNK signaling in intestinal stem cells, causing dys‐homeostasis of their proliferation and differentiation. This impairs intestinal epithelial cell turnover. Thymoquinone in food can protect the intestines from SDS damage by reducing JNK signaling.

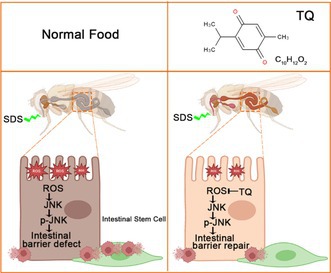

## 1 Introduction

Ulcerative colitis is a kind of inflammatory bowel disease with nonspecific intestinal inflammation and disruption of the intestinal barrier of unknown etiology (Xavier and Podolsky 2007). The incidence of inflammatory bowel disease has been on the rise globally, driven by factors such as poor dietary habits, increased life stress, and changes in the living environment (Olen et al. 2020). Traditional treatment methods for inflammatory bowel disease typically involve a step‐up therapy approach, which includes aminosalicylic acid, glucocorticoids, immunosuppressants, and biological agents (McLean and Cross 2014). However, the efficacy of these conventional treatments is often short‐lived, and long‐term use leads to serious side effects, including infections, osteonecrosis, and malignancy (McLean and Cross 2014). This has underscored the unmet need for developing new treatments that are both safer and more effective.

The search for novel therapies has increased interest in dietary nutrients and natural compounds, which have shown potential in protecting the intestine against external environmental stresses in experimental models of inflammatory bowel disease. Previous investigations revealed that many dietary nutrients, such as *Acanthopanax senticosus* (Huang et al. 2011), *Crocus sativus L*. (Liu et al. 2016), and *Rhodiola crenulata* (Yi et al. 2018), could protect the intestine against external environmental stresses in experimental animal models of ulcerative colitis. *Nigella sativa* (also known as Nigella, black cumin, black caraway, or kalonji) is an annual herb belonging to the family of Ranunculaceae, has been consumed as a spice worldwide and has a history of use in traditional medicine for over 2000 years (Takruri 1998). *Nigella sativa* seeds taste like a mixture of oregano, black pepper, and onion, and have been consumed as a safe spice worldwide (Yimer et al. 2019). They are usually used to flavor vegetables, curry, poultry, bread, and salads. The seeds and oil of *Nigella sativa* possess a range of biological activities, including antimicrobial, antiinflammatory, antioxidant, and anticancer properties, alongside hypoglycemic, spasmolytic, and bronchodilator effects (Tabassum et al. 2018), and have been commonly used as a traditional remedy for various diseases in many countries (Takruri 1998). *Nigella sativa* oil has been reported to decrease tissue damage, oxidative stress, and inflammatory factors in ulcerative colitis mouse models (Genc et al. 2011; Isik et al. 2011). However, the underlying mechanism is still not clear. *Nigella sativa* oil contains many bioactive substances, including thymoquinone (2‐Isopropyl‐5‐methyl‐1,4‐benzo‐quinone, C_10_H_12_O_2_, the main active component), thymohydroquinone, dithymoquinone, and so on (Kooti et al. 2016). Thymoquinone has been a subject of numerous studies due to its diverse biological functions such as antiinflammatory (Ahmad et al. 2020), antitumor (Mahmoud and Abdelrazek 2019), and antibacterial (Chaieb et al. 2011) activities. Recent reports have suggested that thymoquinone can mitigate intestinal damage induced by irradiation in mice by decreasing the apoptosis and modulating the DNA damage in the small intestine (Hou et al. 2018). However, the effect of thymoquinone on ulcerative colitis and the underlying mechanisms remain to be fully understood.

The pathogenesis of inflammatory bowel disease is complex and multifactorial, involving genetic factors, gut microbiota dysbiosis, immune system disorders, and intestinal barrier damage. Current treatments often focus on suppressing excessive immune responses to reduce inflammation. However, recent evidence suggests intestinal barrier damage occurs before the onset of inflammatory bowel disease, allowing intestinal contents to enter the body and activate immune cells, leading to progressive tissue damage and clinical inflammation. During the occurrence and development of the disease, intestinal barrier function continues to be disrupted, and mucosal immune system dysfunction occurs, leading to progressive tissue damage (Zheng and Duan 2023). The intestinal barrier is a multi‐layer structure, including microbial, chemical, mechanical, and immune barriers, which can protect the host from pathogens. In the face of these injury factors, the intestinal cells will die. Still, the intestinal stem cells will continue to produce new cells to supplement and repair the intestinal barrier (Zheng and Duan 2023), so the loss of control of the self‐renewal of intestinal stem cells would lead to ulcerative colitis (Wang, Liu, et al. 2022). Therefore, restoring the integrity of the intestinal epithelium and rescuing the viability of intestinal stem cells are critical therapeutic targets for ulcerative colitis (Watanabe et al. 2022).

The excessive production of reactive oxygen species (ROS) significantly contributes to the advancement of inflammatory bowel disease, thus emerging as a promising target for treatment (Rao et al. 2022). Besides, many signaling pathways contribute to the development of ulcerative colitis, such as the NF‐κB signaling pathway (Lin et al. 2022), the Bcl‐2/Bax signaling pathway (Fu et al. 2022), the c‐Jun N‐terminal kinase (JNK) signaling pathway (Wang, Bai, et al. 2022), and so on. Numerous anti‐oxidant compounds have shown potential in modulating signaling pathways involved in disease development (Samanta et al. 2024). In addition, more and more natural compounds extracted from traditional medicine and food supplements exhibit significant protective effects against diseases through signaling pathways (Wang et al. 2024). However, whether thymoquinone could protect ulcerative colitis through signaling pathways has not been reported. Given the established anti‐oxidant capacity of thymoquinone and its presence in a traditionally used remedy, studying its effects on inflammatory bowel disease, mainly through the modulation of signaling pathways like the JNK pathway, could reveal new avenues for treatment.

Many inflammatory bowel disease models, especially the chemical compounds colitis models, have been used to test the efficacy of newly developed drugs (Baydi et al. 2021). *Drosophila melanogaster* (after this *Drosophila*) is a promising model organism for studying human enteric diseases because of its many advantages (Capo et al. 2019). The intestinal structures and functions of *Drosophila* are similar to mammals (Xiao and Zhou 2016). The activity of intestinal stem cells is identical in *Drosophila* and humans. One intestinal stem cell generates one new intestinal stem cell and one enteroblast through mitosis (Ren et al. 2015). One enteroblast differentiates into one intestinal enterocyte and one enteroendocrine cell, and the enterocytes are responsible for nutritional absorption (Zhou et al. 2015). Therefore, *Drosophila* has become a strength model for studying intestinal stem cells (Petsakou and Perrimon 2023). Moreover, the *Drosophila* ulcerative colitis model generated by a chemical compound sodium dodecyl sulfate (SDS), which induces epithelial cell injury in the intestine, has been widely used to help elucidate the molecular action of many toxicants, drugs, and bioactive ingredients (Ohtsuka and Sanderson 2003; Wei et al. 2022; Zhou et al. 2016).

This study aims to explore the effect of thymoquinone on intestinal injury induced by SDS utilizing the *Drosophila* model and to uncover the underlying mechanisms, focusing on its potential to protect against intestinal damage and regulate key signaling pathways involved in the disease process. The results showed that consuming thymoquinone from the fetal stage can prevent chemical‐induced ulcerative colitis in adulthood by suppressing the JNK signaling pathway based on its antioxidant properties. This research holds substantial importance for enhancing the nutritional and therapeutic properties of thymoquinone in forthcoming applications.

## 2 Experimental Section

### 2.1 Materials and Reagents

Thymoquinone (purity ≥ 98%, #T115128) was provided by Aladdin Co. Ltd. (Shanghai, China). Brilliant blue FCF (#80717) and SDS (#L4509) were from Shanghai Macklin Biochemical Co. Ltd. (Shanghai, China). The phosphate‐buffered saline (PBS, #ST476), 2‐(4‐Amidinophenyl)‐6‐indolecarbamidine dihydrochloride (DAPI, #C1005), the lipid peroxidation (MDA) assay kit (#S0131), catalase (CAT) assay kit (#S0051) and total superoxide dismutase (SOD) assay kit (#S0101) were provided by Beyotime Biotechnology (Shanghai, China). The 2′,7′‐dichlorodihydrofluorescein diacetate (H_2_DCFDA, #D399) was obtained from Thermo Fisher Scientific Co. Ltd. (Shanghai, China). The anti‐phospho‐JNK (pJNK) antibody (#07‐175) and the Cy3‐conjugated goat anti‐rat IgG (#A0521) were from Sigma Aldrich (Shanghai, China).

### 2.2 *Drosophila* Stocks and Culture Medium

The Vienna *Drosophila* RNAi Centre provided wild‐type flies *w* ^*1118*^ (V#60000). *esg*‐GAL4 UAS‐GFP; tub‐GAL80^ts^ (*esg* ^*ts*^>GFP), *Su(H)GBE*‐GAL4 UAS‐GFP; tub‐GAL80^ts^ (*Su(H)GBE* ^ts^>GFP) and *MyO1A*‐GAL4 UAS‐GFP; tub‐GAL80^ts^ (*MyO1A* ^ts^>GFP) flies were kindly provided by Dr. Lihua Jin (Zhang et al. 2020). Vkg.GFP trap (Ke et al. 2018) was from Dr. Jose C. PASTOR‐PAREJA. A dominant negative form of the basket (bsk^DN^, #6409), which could be used as JNK inhibition, was from the Bloomington *Drosophila* Stock Center. Flies were reared on standard cornmeal media at 25°C and 60% humidity with a 12 h light: 12 h dark cycle (Wei et al. 2021).

### 2.3 Fly Collection and Feeding Design

**FIGURE 1:**
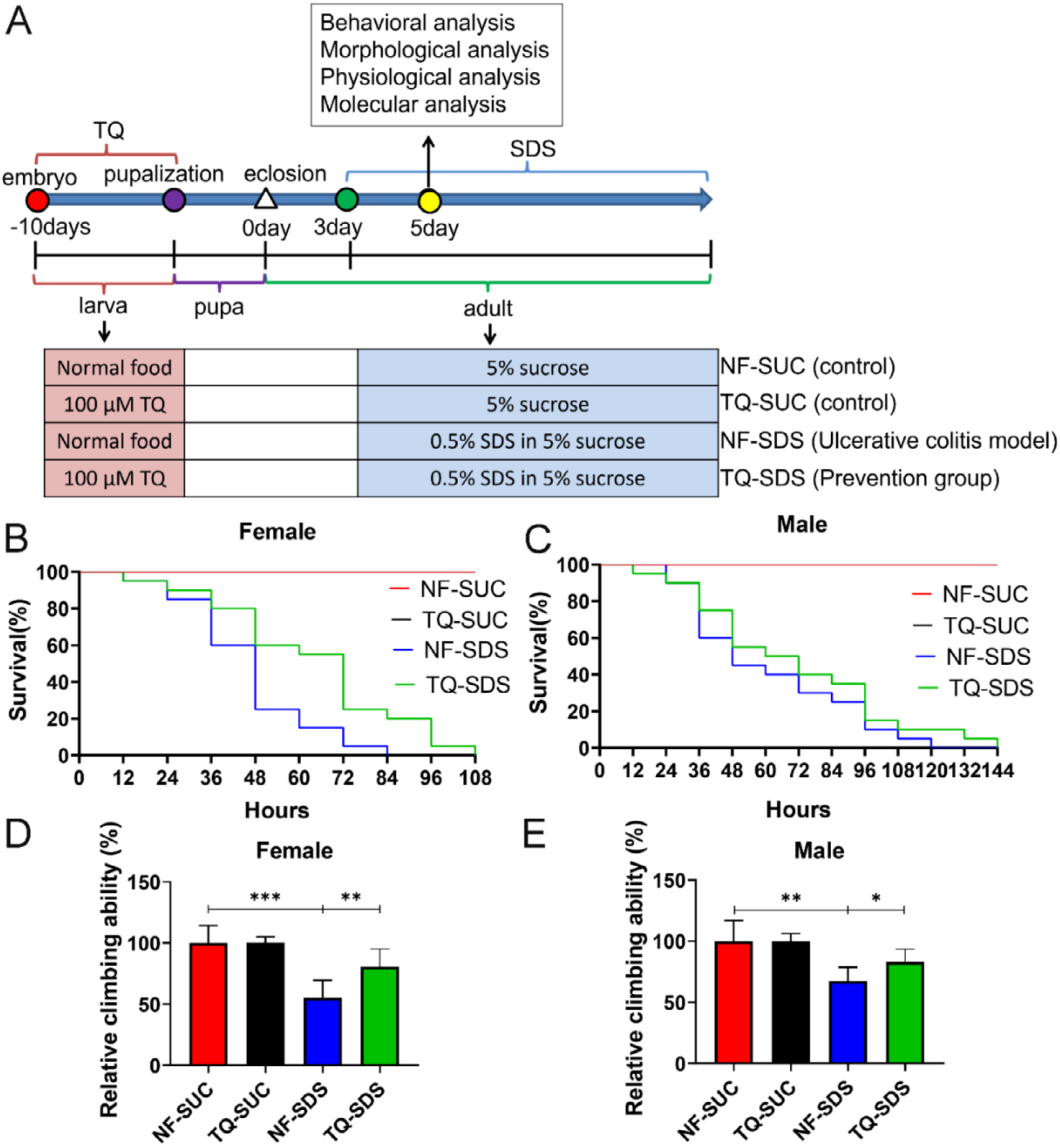
Thymoquinone pretreatment appears to mitigate the decreased the survival and impaired climbing performance in *Drosophila* caused by SDS. (A) Schematic diagram of the experimental design. Numbers in the lateral axis mean days after eclosion (+) or before eclosion (−). (B, C) Pretreatment with thymoquinone was found to ameliorate the diminished survival rates in both female (B) and male (C) groups subjected to 0.5% SDS exposure, which serves as a model for ulcerative colitis (NF‐SDS). Each vial contained 20 larvae, *n* = 6 vials per experimental group. (D, E) The impairments in *Drosophila*'s climbing abilities of both females (D) and males (E) caused by SDS (NF‐SDS) could be alleviated by thymoquinone pretreatment (thymoquinone‐SDS). NF‐SDS, normal food‐SDS; NF‐SUC, normal food‐sucrose; TQ‐SDS, thymoquinone‐SDS; TQ‐SUC, thymoquinone‐sucrose. Each vial contained 20 larvae, *n* = 6–10 vials per experimental group. *p < 0.05, **p < 0.01, and ***p < 0.001.

Crosses were flipped directly on normal food (control) or food containing 100 μM thymoquinone, and then the flies were raised on these mediums from the embryo (−10 days) to adults (0 days) (Figure 1A). Newly eclosed flies were collected within 12 h (0 days adults) and allocated to normal food vials. Approximately 20 single‐sex flies were kept per vial. The flies were cultured on normal food for 3 days and then allocated to vials containing 5% sucrose food (control, NF‐SUC) or 0.5% SDS in 5% sucrose solution food (NF‐SDS and thymoquinone‐SDS) for 48 h to induce acute colitis. The flies were transferred to fresh vials every day. The climbing assay, intestinal morphology assay, smurf assay, ROS assay, immunohistochemistry, and fluorescence microscopy assay were performed at 5 days. Figure 1A outlines the experimental scheme.

### 2.4 Survival Assay

For survival assay, 3‐day newly emerged adults reared on normal food (NF‐SUC and NF‐SDS) or 100 μM thymoquinone (thymoquinone‐SDS) were starved for 2 h and then transferred to tubules containing filter papers hydrated with 5% sucrose (wt/vol) (control, NF‐SUC) or 0.5% SDS in 5% sucrose (wt/vol) (NF‐SDS or thymoquinone‐SDS). 30 females or 30 males were put in each tube. Replace new filter papers and solutions every day. Record the number of dead and surviving flies every day. Data are from three independent experiments.

### 2.5 Climbing Assay

The climbing assay was performed as previously reported (Madabattula et al. 2015; Xiao 2021). Conduct the experiments in ambient light at 25°C and 60% humidity. 10 newly emerged flies (~3 day‐olds) were exposed to sucrose (5%, wt/vol) with or without SDS (0.5%, wt/vol) for 48 h. Then, the flies were collected and placed in a plastic cylinder for 2 h of recovery from CO_2_ exposure. When the climbing assay was performed, all *Drosophila* were gently tapped to the bottom of the plastic cylinder. After 6 s of climbing under red light, the number of *Drosophila* reaching 8 cm was recorded. Data are from three independent experiments.

### 2.6 Intestinal Morphology Assay

For the morphology assay, 10 female adults were dissected in 1 × PBS for microscopic observation of the intestinal morphology. For the fluorescent assay, BM‐40‐SPARC‐GAL4 was crossed to the Vkg.GFP trap; 6 progeny female adults were dissected in 1 × PBS and mounted with 60% glycerol. A Nikon ECLIPSE Ti2‐U microscope (Nikon, Tokyo, Japan) was used to observe and take pictures.

### 2.7 Smurf Assay and Smurf Ness Assay

Food was prepared using the standard medium with 2.5% (wt/vol) Brilliant Blue FCF supplementation. The daily experiment operation is as follows: for 4 days, newly emerged females reared on normal food (NF‐SUC and NF‐SDS) or 100 μM thymoquinone (thymoquinone‐SDS) were starved for 2 h and then treated with a 5% sucrose solution (NF‐SUC) or 0.5% SDS in a 5% sucrose solution (NF‐SDS or thymoquinone‐SDS) for 6 h. Then, the *Drosophila* have been transferred to Brilliant Blue FCF‐dyed food for 16 h. The *Drosophila* were frozen to death for morphological analysis, and pictures were taken using a Nikon stereomicroscope. For the smurfness, the *Drosophila* were homogenized in 1 × PBS after 7 days, and the absorbance at 625 nm of the resulting centrifugal supernatant was determined.

### 2.8 Reactive Oxygen Species Assay

After feeding with SDS (0.5%, wt/vol) for 48 h, 15 *Drosophila* were dissected in 1 × PBS. Then, the intestines were incubated in 5 μM H_2_DCFDA for 10 min. After that, the intestines were washed 3 times with 1 × PBS, then observed and photographed with a Nikon ECLIPSE Ti2‐U microscope (Nikon, Tokyo, Japan). The experiments were conducted using an ice bath to maintain a low‐temperature environment. The experiment was repeated three times.

### 2.9 Immunohistochemistry and Fluorescence Microscopy Assay

The intestines of 5 ‐ day ‐ old females were dissected out and fixed with 4% paraformaldehyde for 10 min. Then, the intestines were stained and mounted with the following antibodies: rabbit anti‐phosphorylated c‐Jun (pJNK) (1:200, Cat#07‐175, Sigma‐Aldrich Trading Co. Ltd). The secondary antibody was Cy3‐conjugated goat anti‐rat IgG (1:500, #A0521, Beyotime Biotechnology).

The intestines were dissected and fixed with 4% paraformaldehyde at room temperature for 10 min. Then, the intestines were washed three times with 0.3% PBST. For DAPI staining, the intestines were incubated with 50 ng/mL DAPI at room temperature for 8 min. The results were observed and taken with a Zeiss LSM710 Meta confocal microscope. ImageJ was used to calculate the pJNK levels. Intestines of *esg* ^*ts*^>GFP, *Su(H)* ^*ts*^>GFP, and *MyO1A* ^*ts*^>GFP flies were dissected, stained, mounted, and taken as mentioned above. The GFP intensity in posterior midguts of *esg* ^*ts*^>GFP, *Su(H)* ^*ts*^>GFP, and *MyO1A* ^*ts*^>GFP was quantified, and the fluorescence of GFP+ cells was measured using ImageJ.

### 2.10 Statistical Analysis

Each experimental procedure was conducted a minimum of three times to ensure reliability. For statistical analysis and data visualization, GraphPad Prism software was utilized. A Student's *t*‐test was employed to compare data between two groups, while a one‐way ANOVA was applied to assess differences among several groups. The results were depicted as the mean value with standard error of the mean (SEM). Significance levels are denoted by asterisks (*p < 0.05, **p < 0.01, and ***p < 0.001).

## 3 Results

### 3.1 Thymoquinone Protects *Drosophila* From Sodium Dodecyl Sulfate (SDS)‐Induced Damage

To investigate the effect of thymoquinone on intestinal homeostasis, *Drosophila* were fed 100 μM thymoquinone from the embryonic stage (Figure 1A). Therefore, these *Drosophila* consumed thymoquinone in their diet throughout the larval stage (Figure 1A). We found that concentrations below 100 μM thymoquinone did not yield significant effect on the model, while concentrations above this level adversely affected the normal growth and development of *Drosophila*. Based on a balance between efficacy and safety, 100 μM thymoquinone was chosen. Regarding intestinal health, locomotor ability, and lifespan, our data indicate that 100 μM thymoquinone does not negatively impact these parameters in normal *Drosophila*. Importantly, 100 μM thymoquinone effectively rescued the phenotypes associated with colitis in this model, demonstrating its potential therapeutic value without compromising the well‐being of healthy flies. After enclosing, the *Drosophila* adults were exposed to 0.5% SDS, a chemical disrupting the epithelium, to establish the ulcerative colitis model (NF‐SDS). Adult females fed with sucrose were used as the control. This ulcerative colitis model displayed a decreased survival rate and reduced climbing activity, as previously validated (Figure 1B,C) (Wei et al. 2022). As shown in Figure 1B,C, pretreatment with thymoquinone (thymoquinone‐SDS, prevention group) exhibited extended longevity (Figure 1B,C). The improvement effect was more substantial in females, with 38% and 29% increases for the medium and the maximum lifespans of NF‐SDS, respectively (Figure 1B). A dramatic improvement effect was also exhibited in males (Figure 1C). After treatment with SDS for 48 h, the climbing ability of females and males was significantly reduced to 55% and 68%, respectively (Figure 1D,E). Consistent with the results described above, pretreatment with thymoquinone significantly improved the reduced climbing ability (26% increase in females; 15% increase in males) (Figure 1D,E). Considering that the NF‐SDS females and males exhibited largely similar phenotypes, we showed results of females hereafter. The findings indicate that thymoquinone pretreatment may offer protection against ulcerative colitis triggered by SDS.

### 3.2 Thymoquinone Can Restore the Disrupted Intestinal Epithelial Barrier of Ulcerative Colitis

**FIGURE 2:**
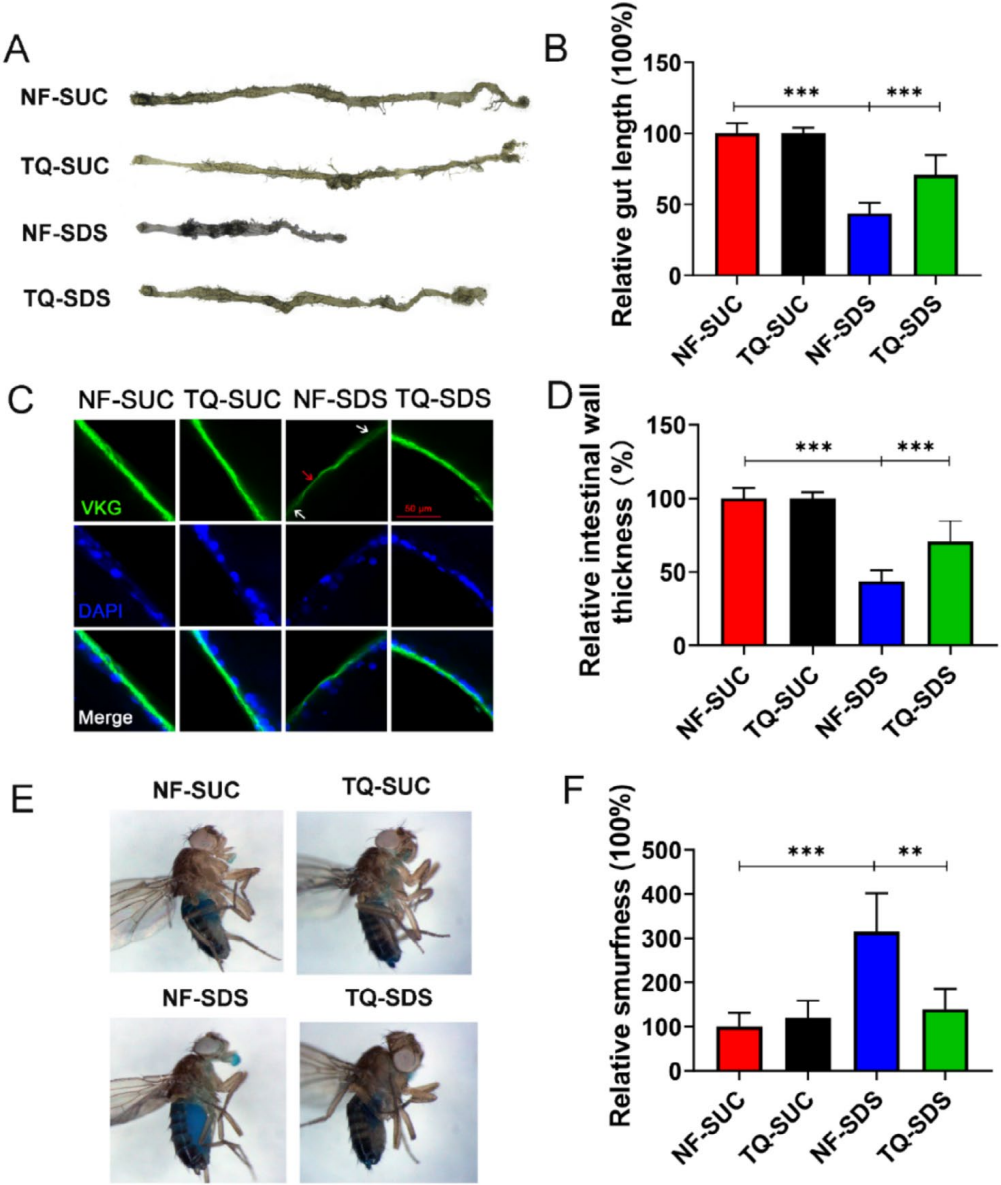
Thymoquinone pretreatment could alleviate the deterioration of the intestinal structure and the breakdown of the barrier's integrity caused by SDS. (A) The shortened gut length and thickened intestines of *Drosophila* exposed to 0.5% SDS for 48 h were significantly alleviated by thymoquinone pretreatment. *n* = 6–10 replicates per group. (B) Quantitative measurement of the gut length in (A). (C) GFP‐Vkg (green) accumulates in the intestinal wall. SDS damages the integrity (white arrow) and thickness (red arrow) of the intestinal wall, which were restored by thymoquinone pretreatment. (D) Quantitative measurement of the thickness of the intestinal wall in (C). (E) Utilization of the “Smurf” experiment to assess *Drosophila* gut integrity. The amount of blue pigment in the body presents the quantification of gut barrier defect phenotype in *Drosophila*, emphasizing the protective role of thymoquinone in maintaining gut barrier integrity. *n* = 6–10 replicates per group. (F) The permeability of the intestinal barrier was assessed by quantifying the Smurfness levels. NF‐SDS, normal food‐SDS; NF‐SUC, normal food‐sucrose; TQ‐SDS, thymoquinone‐SDS; TQ‐SUC, thymoquinone‐sucrose. *n* = 6–10 replicates per group. *p < 0.05, **p < 0.01, and ***p < 0.001.

SDS‐induced gut length reduction, a marker for intestinal damage, was observed in *Drosophila* (Figure 2A,B). As shown in Figure 2A,B, there was a significant reduction (~44% of control) in the gut length of the NF‐SDS group. But in the thymoquinone pretreatment group, recovery of gut length (from ~44% to 72%) was observed (Figure 2A,B). Besides, SDS induction can lead to abnormal intestinal thickening in *Drosophila*, which thymoquinone pretreatment can also improve (Figure 2A,B). The severity of colitis in ulcerative colitis model was further supported by histopathological analysis of the intestine (Figure 1C,D). Viking (Vkg, α2 chain) is a *Drosophila* Collagen IV component that could mark basement membrane (Natzle et al. 1982). Then Vkg.GFP trap (Ke et al. 2018) was used to detect the intestinal wall. In contrast to the control, the intestines of the NF‐SDS group exhibited heightened disease severity, characterized by destruction and thinning of the intestinal walls (Figure 1C,D). Consistent with the above data, these defects were restored in the thymoquinone pretreatment group (Figure 1C,D). The intestinal epithelial barrier could be disrupted by SDS (Wei et al. 2022). The Smurf experiment, which stands for “Smurf‐related uptake and response to food” is a method used to assess the intestinal integrity of *Drosophila melanogaster* . The chemical‐induced intestinal epithelial barrier could be detected by a nonabsorbable blue food dye, Brilliant Blue FCF (Jin et al. 2020). The Smurf experiment utilizes the uptake of Brilliant Blue FCF by the gut to evaluate the permeability and health of the fruit fly's intestinal barrier. Compared with the control, the blue distribution increased after SDS treatment, showing a 215% increase in Smurfness (Figure 2E,F). Pretreatment with thymoquinone reduced the blue distribution, and the phenotype was similar to that of the control. The findings suggest that thymoquinone helps maintain the integrity of the intestinal epithelial barrier, thus safeguarding against ulcerative colitis.

### 3.3 Thymoquinone Regulates the Proliferation and Differentiation of Intestinal Stem Cells

**FIGURE 3:**
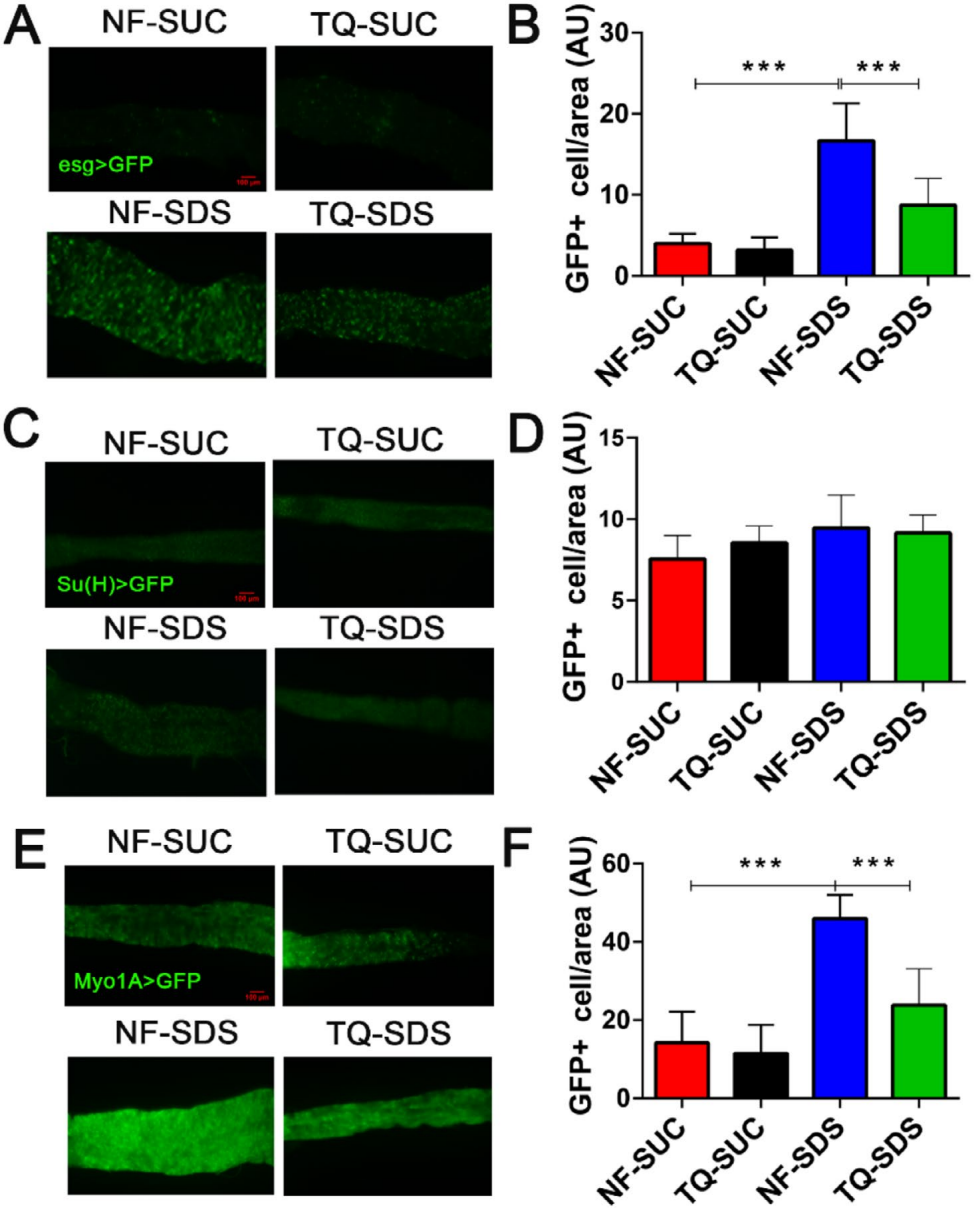
Pretreatment with thymoquinone shielded the intestines against the proliferation and differentiation of intestinal stem cells caused by SDS. (A) Thymoquinone pretreatment notably decreased the SDS‐induced proliferation and differentiation of intestinal stem cells and enteroblasts. Intestinal stem cells and enteroblasts were marked by esg‐Gal4^ts^>GFP. *n* = 10 replicates per group. (B) The fluorescence levels of GFP‐positive intestinal stem cells and enteroblasts in (A). (C) The increased enteroblasts in the intestines of the ulcerative colitis model (NF‐SDS) could be decreased by thymoquinone pretreatment. Enteroblasts were marked by Su(H)‐Gal4^ts^>GFP. *n* = 10 replicates per group. (D) The intensity of GFP fluorescence in enteroblasts within the sections in (C). (E) The increased enterocytes in the intestines of the ulcerative colitis model (NF‐SDS) could be decreased by thymoquinone pretreatment. Enterocytes were marked by Myo1A‐Gal4^ts^>GFP *n* = 10 replicates per group. (F) The intensity of GFP fluorescence in enteroblasts within the sections in (E). NF‐SDS, normal food‐SDS; NF‐SUC, normal food‐sucrose; TQ‐SDS, thymoquinone‐SDS; TQ‐SUC, thymoquinone‐sucrose. *p < 0.05, **p < 0.01, and ***p < 0.001.

The growth and maturation of intestinal stem cells play a pivotal role in maintaining the integrity of the intestinal epithelial barrier (Santos et al. 2018). Each division of a multipotent intestinal stem cell forms another intestinal stem cell and an enteroblast daughter cell. This enteroblast then matures into either an enterocyte or an enteroendocrine cell (Zeng and Hou 2015). To delve deeper into thymoquinone's impact on intestinal epithelial damage caused by SDS, we examined the processes of intestinal stem cell proliferation and differentiation (Figure 3). The numbers of intestinal stem cells/enteroblasts could be identified through the expression of UAS‐GFP under the control of esg‐Gal4^ts^ (esgGal4^ts^>UAS‐GFP). This was indicated by the fluorescence intensity of GFP‐positive cells (Nilangekar et al. 2019). Adult females were fed sucrose (Suc, control) or SDS, and their intestines were dissected for analysis. Figure 3A,B illustrate that, in contrast to the control group, the NF‐SDS group exhibited a substantial 308% increase in the fluorescence intensity of GFP‐tagged intestinal stem cells and enteroblasts (Figure 3B), similar to what was reported previously (Amcheslavsky et al. 2009; Tian et al. 2015). The proliferation of intestinal stem cells and enteroblasts, which had increased with SDS treatment, was notably reduced by thymoquinone (down 49%). This suggests that the induced intestinal stem cell mitosis caused by SDS was blocked by thymoquinone pretreatment. To further differentiate intestinal stem cell proliferation and differentiation, the enteroblasts‐driver *Su(H)(Suppressor of Hairless)* ^ts^>GFP flies (Zeng et al. 2010) were used to characterize enteroblasts (Figure 3C,D). However, the number of enteroblasts was not affected by SDS or thymoquinone (Figure 3C,D). Then, the enterocytes were marked by the expression of UAS‐GFP driven by the enterocyte‐specific Gal4 (MyO1‐Gal4^ts^) (MyO1A>GFP) (Figure 3E,F). In adult female flies treated with SDS, the count of GFP‐expressing enterocytes in the midgut was markedly higher than in the control group (Figure 3E,F). However, thymoquinone pretreatment alleviated the abnormally increased enterocyte number (Figure 3E,F). Taken together, the intestinal stem cell division could be triggered by tissue damage in *Drosophila*, and lifelong thymoquinone consumption from childhood into adulthood may safeguard the adult intestines against SDS‐induced injury.

### 3.4 Thymoquinone Maintains the Intestinal Barrier by Reducing the Intracellular Oxidative Stress

**FIGURE 4:**
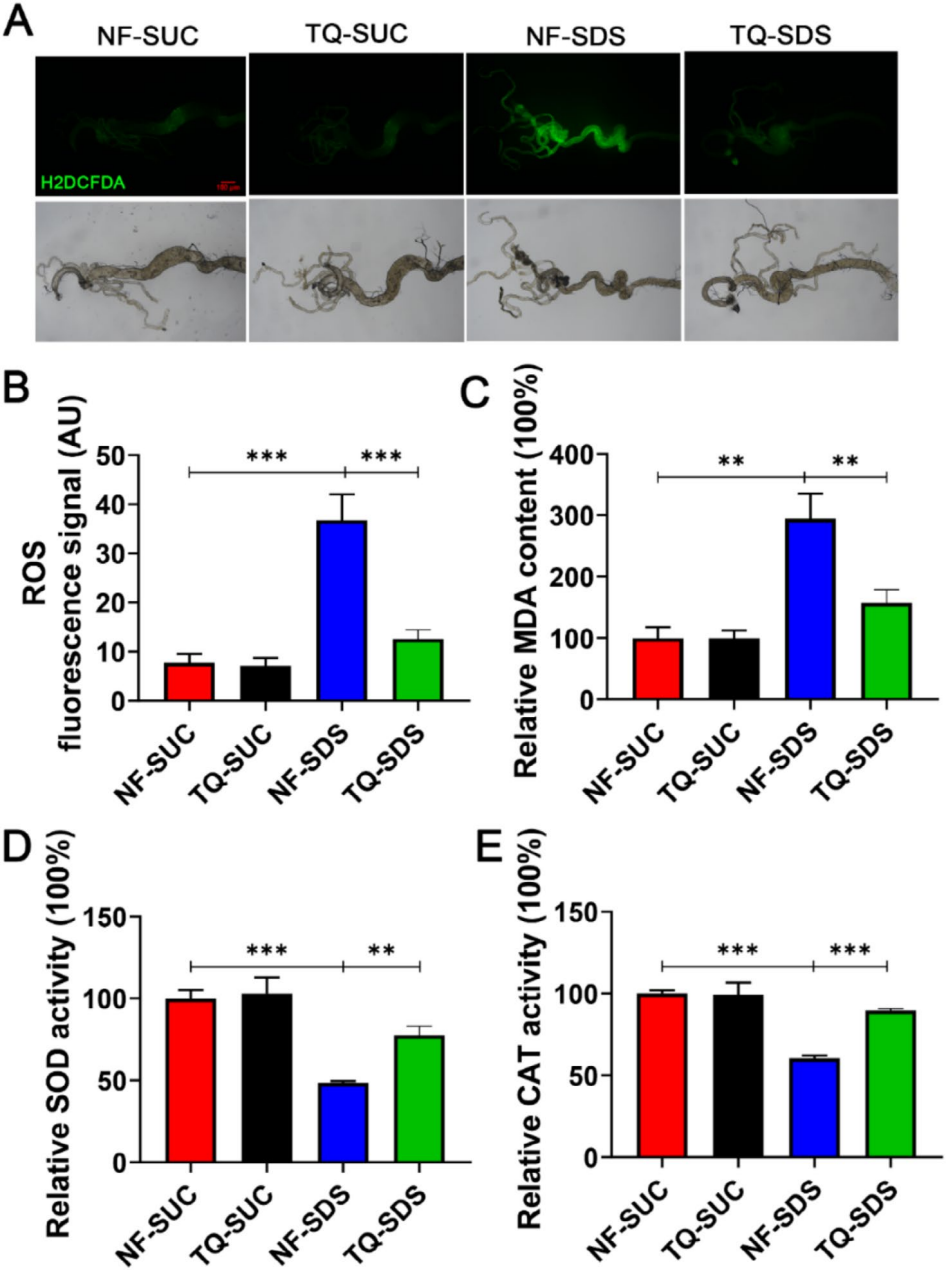
Thymoquinone pretreatment alleviated SDS‐induced oxidative stresses in the intestines. (A) Thymoquinone shielded epithelial cells from oxidative stress triggered by SDS. This was demonstrated by H_2_DCFDA staining in the anterior midgut of female flies following a 48‐h exposure to 0.5% SDS. *n* = 10 replicates per group. (B) Fluorescence intensity of Reactive Oxygen Species (ROS) levels in (A). (C) Pretreatment with thymoquinone reduced the elevated malondialdehyde (MDA) levels induced by SDS. (D, E) Thymoquinone pretreatment enhanced the activity levels of total superoxide dismutase (SOD) and catalase (CAT) following a 48‐h SDS exposure. NF‐SDS, normal food‐SDS; NF‐SUC, normal food‐sucrose; TQ‐SDS, thymoquinone‐SDS; TQ‐SUC, thymoquinone‐sucrose. *n* = 6 replicates per group. *p < 0.05, **p < 0.01, and ***p < 0.001.

ROS overproduction was previously observed in chemical‐induced intestines (Balmus et al. 2016). Cellular oxidative stress is the main reason for the decline of tissue homeostasis and functional degradation (Xie et al. 2019). Overproduction was previously observed in chemical‐induced intestines (Balmus et al. 2016). Cellular oxidative stress is the main reason for the decline of tissue homeostasis and functional degradation (Xie et al. 2019). Next, we investigated the mechanism by which thymoquinone protects against SDS‐induced injury. ROS generation in the intestinal epithelium is an important manifestation of ulcerative colitis (Chen et al. 2020). ROS have emerged as central regulators of intestinal stem cell function (Morris and Jasper 2021). Therefore, we wondered whether the regulation of thymoquinone on SDS resistance is related to ROS. We used H_2_DCFDA, a cell‐permeable probe (Kalyanaraman et al. 2012), to ascertain the intracellular ROS concentration. As shown in Figure 4A,B, in the ulcerative colitis model, adult female posterior midgut exhibited a significant increase in ROS levels compared to control, and the over‐produced ROS was attenuated significantly in thymoquinone‐SDS (66% decrease in fluorescence signal, compared with the NF‐SDS group). The lipid peroxidation could be indicated by the malondialdehyde (MDA) content. The results showed that SDS exposure significantly increased the MDA content by 194%, which could be alleviated by thymoquinone to 57% (Figure 4C). The findings indicate that thymoquinone protected the intestinal barrier of ulcerative colitis flies by reducing intracellular oxidative stress.

Numerous antioxidant enzymes, such as superoxide dismutase (SOD) and catalase (CAT), shield organisms against oxidative stress (Buzdagli et al. 2022). As shown in Figure 4D,E, the activity of both SOD and CAT was dramatically reduced in NF‐SDS (reduced by 52% and 40%, respectively). The reduced activity of SOD and CAT caused by SDS was restored by pretreatment with thymoquinone (increased by 29% and 30%, respectively). Altogether, these results suggested that pretreatment with thymoquinone can prevent the gut function damage and maintain intestinal barrier and homeostasis by reducing intracellular oxidative stress.

### 3.5 JNK Signaling in Intestinal Stem Cells Is Involved in Thymoquinone's Protective Effect Against SDS‐Induced Intestinal Damage

**FIGURE 5:**
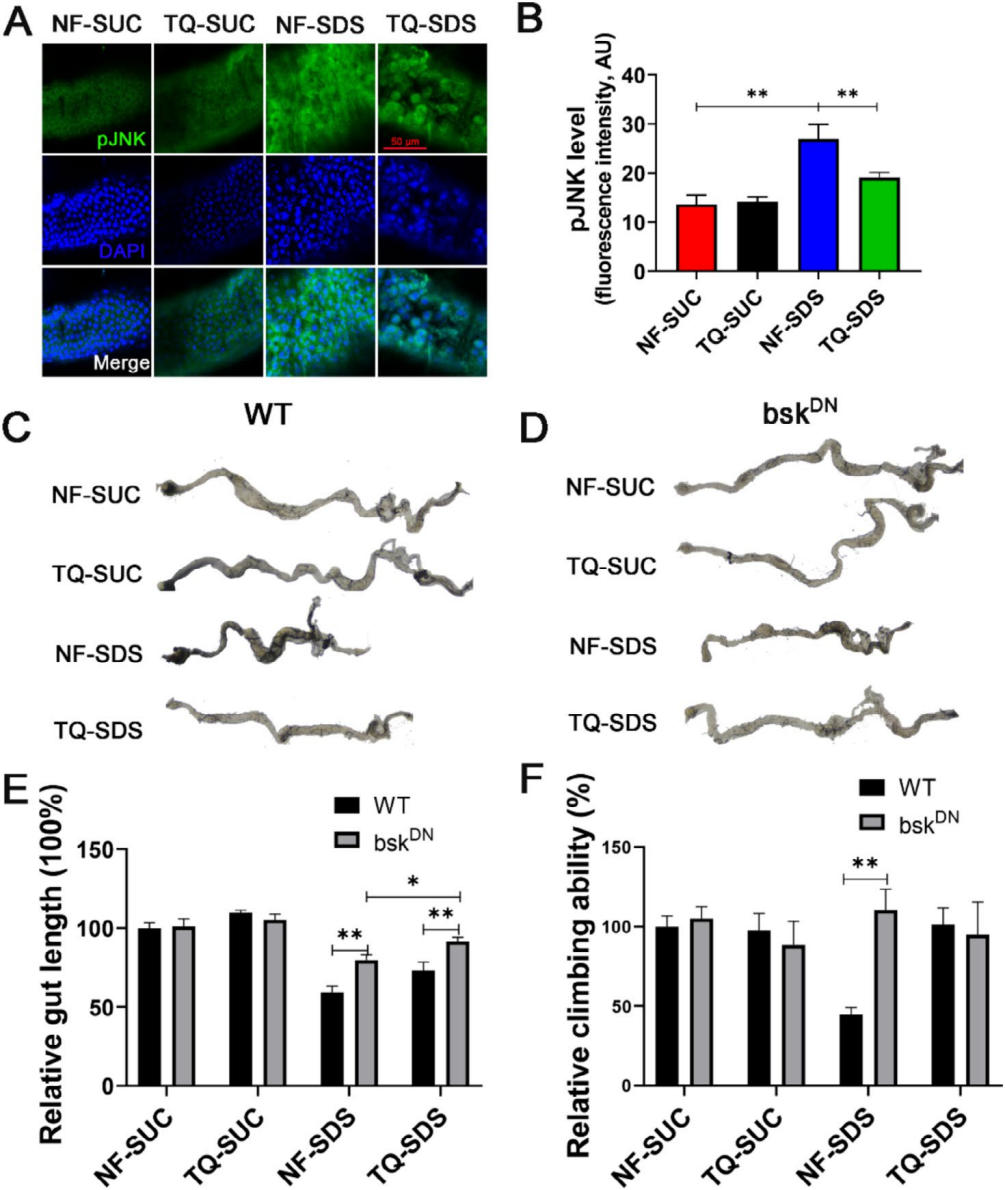
The c‐Jun N‐terminal kinase (JNK) signaling pathway played a role in thymoquinone's protective effect against ulcerative colitis. (A) The JNK activation induced by SDS was significantly attenuated by thymoquinone. *n* = 6 replicates per group. (B) Quantitative measurement of (A). (C) Intestinal morphology of *Drosophila* exposed to 0.5% SDS for 48 h. (D) The shortened gut length and thickened intestines of *Drosophila* exposed to 0.5% SDS for 48 h were significantly alleviated by JNK inhibition. *n* = 6–10 replicates per group. (E) Quantitative measurement of the gut length in (D). (F) The climbing defects of *Drosophila* exposed to 0.5% SDS for 48 h were significantly alleviated by JNK inhibition. *n* = 6 replicates per group. NF‐SDS, normal food‐SDS; NF‐SUC, normal food‐sucrose; TQ‐SDS, thymoquinone‐SDS; TQ‐SUC, thymoquinone‐sucrose. *p < 0.05, **p < 0.01, and ***p < 0.001.

Considering that the JNK signaling pathway plays a role in ROS‐driven proliferation and differentiation of intestinal stem cells (Santabárbara‐Ruiz et al. 2015), we wondered whether the protection of thymoquinone on intestine injury involves the JNK signaling. We next examined the expression of activated JNK (phosphorylated‐JNK, pJNK) in the intestine by immunohistochemical staining. Our findings revealed a 1.98‐fold increase in pJNK levels within the NF‐SDS group, compared with NF‐SUC (Figure 5A,B). The increased pJNK level was restored by 29% in thymoquinone‐SDS (Figure 5A,B). The findings suggest that thymoquinone's protective role in ulcerative colitis is associated with the JNK signaling pathway.

To further confirm whether JNK signaling mediates the protection of thymoquinone on ulcerative colitis, we examined whether JNK inhibition (bsk^DN^) could alter the sensitivity of *Drosophila* to SDS. bsk^DN^ was driven in *Drosophila* intestinal stem cells by esg‐Gal4^ts^. We found that bsk^DN^ does not affect the gut length and climbing activity of wild‐type *Drosophila* fed with sucrose or thymoquinone. However, *Drosophila* with bsk^DN^ can resist intestinal damage and reduced climbing ability caused by SDS. These data suggest that thymoquinone shields the intestine from SDS‐induced damage by dampening JNK signaling in intestinal stem cells.

## 4 Discussion

Over recent years, dietary and functional food options have become increasingly viable for managing inflammatory bowel disease due to their safety, effectiveness, and bioavailability (Mijan and Lim 2018; Shin and Lim 2020). The biotech and agri‐food sectors are crucial in integrating bioactive compounds into everyday diets (Mijan and Lim 2018; Shin and Lim 2020). The intake of plant‐derived foods is especially linked to a lower incidence of inflammatory bowel disease (Shin and Lim 2020). The present study reveals the protective effects of thymoquinone against sodium dodecyl sulfate (SDS)‐induced intestinal damage in *Drosophila melanogaster* , a model that closely mirrors human ulcerative colitis. The potential of thymoquinone to reduce oxidative stress and modulate the JNK signaling pathway suggests a dual role in both managing inflammation and promoting intestinal health, which could be harnessed through dietary interventions.

Thymoquinone has garnered growing interest due to its diverse biological properties (Aslani et al. 2024). The intestinal epithelium plays a crucial role in preserving the balance within the intestines (Okumura and Takeda 2018). Various external pressures, including chemical injury (Antunes et al. 2011), mechanical pressure (Gayer and Basson 2009), and irradiation (Qu et al. 2020), resulted in damage to the intestinal barrier, thus destroying intestinal homeostasis (Schoultz and Keita 2020). It is well known that the self‐renewal ability of intestinal epithelial cells plays an essential role in maintaining intestinal homeostasis, blocking intestinal microbial invasion, and repairing damage. The function of thymoquinone in inflammatory bowel disease has been investigated (Alaaeldin et al. 2023; Lei et al. 2012; Venkataraman et al. 2021). However, its impact on intestinal stem cells has not been the subject of dedicated research. The significant reduction in ROS and the restoration of antioxidant enzyme activities (SOD and CAT) in the presence of thymoquinone indicate its robust antioxidant properties. This is particularly significant as oxidative stress is a driving factor in inflammatory bowel disease, contributing to tissue damage and inflammation. By targeting ROS, thymoquinone may offer a novel therapeutic approach to manage inflammatory bowel disease symptoms and potentially slow disease progression. Moreover, the modulation of the JNK pathway by thymoquinone is a critical finding, as this pathway is implicated in various inflammatory and stress responses. The inhibition of JNK signaling by thymoquinone suggests a mechanism by which it could reduce inflammation and promote intestinal epithelial homeostasis.

Most previous ulcerative colitis studies focused on inflammation, and nuclear factor‐κB (NF‐κB) signaling is the most investigated pathway (Duan et al. 2022; Lin et al. 2022). Besides, other signaling pathways containing Wnt, Bcl‐2/Bax signaling, and Keap1/Nrf‐2 signaling have also been reported to involve this disease (Fu et al. 2022). In addition, we reported that ursolic acid protects the SDS induced *Drosophila* ulcerative colitis model by reducing the JNK/JAK/STAT pathway (Wei et al. 2022). Here, we found that the protective effect of thymoquinone on ulcerative colitis is related to intestinal stem cell proliferation and differentiation. Cells' self‐replication and renewal could repair tissue injury, and intestinal stem cells can self‐renewal (Jiang et al. 2016). Moreover, we found that JNK signaling is involved in SDS‐induced damage. Intriguingly, thymoquinone alleviated the ulcerative colitis process by reducing the JNK signaling. This is the first time we have reported thymoquinone's effect on JNK signaling. Whether other signaling pathways are involved in the protection of thymoquinone on ulcerative colitis remains a further investigation. Altogether, this study indicated that thymoquinone could visibly protect intestinal epithelial homeostasis from chemicals‐induced injury via the JNK‐dependent pathway. Our results point towards a complex interplay between thymoquinone, oxidative stress, and the JNK pathway. The reduction of ROS by thymoquinone likely contributes to the suppression of JNK signaling, affecting the proliferation and differentiation of intestinal stem cells. This mechanism could be crucial in maintaining the integrity of the intestinal barrier and preventing the translocation of luminal content into the mucosa, a hallmark of inflammatory bowel disease. Understanding these mechanisms could lead to the development of targeted therapies that harness the antioxidant and antiinflammatory properties of thymoquinone. This research provides valuable in vivo evidence on the therapeutic effects of thymoquinone in ulcerative colitis, a ROS‐related inflammatory bowel disease that shares molecular biology aspects with Crohn's disease and other ROS‐associated conditions. Our findings suggest that thymoquinone's potential as a drug candidate or food additive for managing not only ulcerative colitis but also other inflammatory bowel diseases and ROS‐related disorders warrants further exploration. Given that dysregulation of the JNK signaling pathway is implicated in a broad range of diseases (Yan et al. 2024), including metabolic disorders, neurological conditions, and cancer, targeting this pathway could offer a promising therapeutic strategy. The possibility that thymoquinone may modulate these diseases through the JNK signaling pathway is a hypothesis that merits additional research.

Future studies should explore thymoquinone's potential as a functional food or therapeutic agent across this spectrum of conditions, potentially expanding its applications beyond inflammatory bowel disease treatment. Although our study provides preliminary evidence for the protective effects of thymoquinone in an inflammatory bowel disease model, further research is needed to translate these findings into practice. Future studies should focus on optimizing the delivery and bioavailability of thymoquinone through food formulations, investigating its synergistic effects with other dietary components, assessing safety profiles, and conducting clinical trials to establish its efficacy in managing inflammatory bowel disease. Additionally, exploring personalized nutrition strategies based on individual genetic and microbiome profiles, conducting large‐scale epidemiological studies, and developing educational programs for consumer awareness will be crucial steps towards integrating thymoquinone as a functional food component for inflammatory bowel disease management and prevention. These future research directions outlined will significantly enhance our comprehension of thymoquinone's therapeutic role within food science and pave the way for innovative food‐based strategies to manage inflammatory bowel disease. By concentrating on these strategic areas, subsequent studies can leverage our findings to transform thymoquinone's potential into tangible dietary interventions for inflammatory bowel disease management.

## 5 Conclusion

**FIGURE 6:**
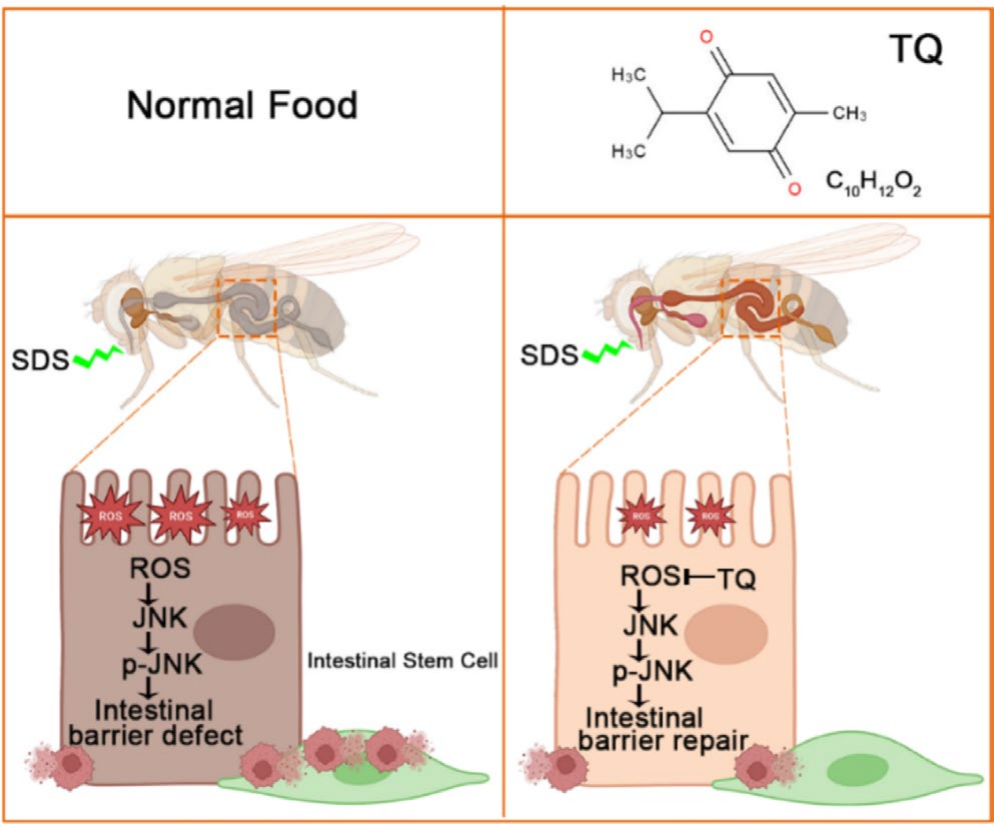
A model to explain the effect of thymoquinone on ulcerative colitis. The ROS induced by SDS in the intestines could activate the JNK signaling pathway in intestinal stem cells. These stresses lead to an imbalance in the proliferation and differentiation of intestinal stem cells. Therefore, the intestinal epithelium fails to undergo rapid cell turnover to maintain the integrity of the intestinal epithelial barrier. Incorporating thymoquinone into the diet may shield the intestines against SDS‐induced harm by modulating the JNK signaling in intestinal stem cells.

In summary, the ulcerative colitis phenotypes of *Drosophila* exposure to SDS, containing behavioral defects and intestinal morphological defects, could be alleviated by pretreatment with thymoquinone. Thymoquinone pretreatment mitigated ROS generation in the intestine and curbed the excessive proliferation and differentiation of intestinal stem cells triggered by SDS. Moreover, the protective effect of thymoquinone against SDS‐induced injury is attributed to the downregulation of the JNK signaling pathway (Figure 6). These findings open up new avenues for research into the role of natural compounds in managing inflammatory bowel disease and underscore the need for further exploration into the mechanisms and applications of thymoquinone.

## Author Contributions

**Kaitong Jia:** data curation (equal), formal analysis (equal), investigation (equal), methodology (equal), validation (equal), visualization (equal), writing – original draft (equal), writing – review and editing (supporting). **Lei Wu:** data curation (equal), formal analysis (equal), investigation (equal), methodology (equal), validation (equal), visualization (equal), writing – original draft (equal), writing – review and editing (supporting). **Ziru Li:** data curation (supporting), formal analysis (supporting), investigation (supporting), methodology (supporting), validation (supporting), writing – review and editing (supporting). **Tian Wei:** data curation (supporting), formal analysis (supporting), validation (supporting), writing – original draft (supporting), writing – review and editing (supporting). **Tingting Fan:** investigation (supporting), methodology (supporting), supervision (supporting), writing – review and editing (supporting). **Guiran Xiao:** conceptualization (lead), funding acquisition (lead), project administration (lead), supervision (lead), visualization (supporting), writing – review and editing (lead).

## Conflicts of Interest

The authors declare no conflicts of interest.

## Data Availability

The data underlying this article will be shared at a reasonable request by the corresponding author.
